# Changing Behavior among Nurses to Track Indwelling Urinary Catheters in Hospitalized Patients

**DOI:** 10.1155/2013/405041

**Published:** 2013-03-06

**Authors:** Bona Yoon, Samantha D. McIntosh, Leslie Rodriguez, Alma Holley, Charles J. Faselis, Angelike P. Liappis

**Affiliations:** ^1^Medical Service, Veterans Affairs Medical Center, 50 Irving Street, NW 4A155, Washington, DC 20422, USA; ^2^Department of Medicine, The George Washington University, Washington, DC 20037, USA; ^3^Nursing Services, Veterans Affairs Medical Center, Washington, DC 20422, USA

## Abstract

Catheter-associated urinary tract infections (CAUTIs) are preventable complications of hospitalization. An interdisciplinary team developed a curriculum to increase awareness of the presence of indwelling urinary catheters (IUCs) in hospitalized patients, addressed practical, primarily nurse-controlled inpatient risk-reduction interventions, and promoted the use of the IUC labels (“tags”). Five thirty-minute educational sessions were cycled over three daily nursing shifts on two inpatient medical floors over a 1-year period; participants were surveyed (*n* = 152) to elicit feedback and provide real-time insight on the learning objectives. Nurse self-reported IUC tagging was early and sustained; after the IUC tag was introduced, there was a significant increase in tagging reported by the end of the block of educational sessions (from 46.2% to 84.6%, *P* = 0.001). Early engagement combined with a targeted educational initiative led to increased knowledge, changes in behavior, and renewed CAUTI awareness in hospitalized patients with IUCs. The processes employed in this small-scale project can be applied to broader, hospitalwide initiatives and to large-scale initiatives for healthcare interventions. As first-line providers with responsibility for the placement and daily maintenance of IUCs, nurses are ideally positioned to implement efforts addressing CAUTIs in the hospital setting.

## 1. Introduction

The presence of an indwelling urinary catheter (IUC) is the principal risk factor for catheter-associated urinary tract infection (CAUTI) development. Despite the risk of prolonged catheter placement, few hospitals actively track catheterized patients, and providers are often not aware of the presence of catheters in their patients [1–3]. 

Nurses are at the frontline of catheter care. As the providers most involved with IUCs in hospitalized patients, nurses are responsible for IUC placement, day-to-day catheter management, and the removal of IUCs. Responsible for specimen collection, nurses play a vital role in the diagnosis of CAUTIs. Among catheterized patients, they are often the first to notice a clinical change or technical problem. 

Despite their central role in IUC care and management, only a handful of published reports have highlighted the role of nurses in the prevention of CAUTI. The majority of these publications have followed a quality improvement (QI) approach, at times bundled with hospital-wide policy changes [4–7]. 

In a recent paper by Drekonja et al. an Internet survey demonstrated inconsistent catheter-related knowledge among nurses [6]. However, reeducation of surgical nurses in urinary catheter management has shown to have a modest decrease in catheter days [7]. Gaps in knowledge may potentially impair the effectiveness nurses may play in the prevention of catheter-related complications. 

With a goal of increasing awareness about the presence of IUCs among medical inpatients, our team developed an educational curriculum. After eliciting staff engagement by cultivating champions and involving the staff in all stages of the process, we instituted a practical, catheter-care curriculum which introduced QI concepts and incorporated principles of basic microbiology and hand hygiene to mirror established infection-control practices. The curriculum targeted aspects of catheter care within the scope of practice of nursing providers and was built on published guidelines [8–10]. 

The purpose of the QI project was to improve compliance with documentation of indwelling urinary catheter insertion. As a tangible application of their education, we promoted the use of the catheter labels (“tags”) and monitored engagement over the course of the educational sessions.

## 2. Methods

Nurses of the medical inpatient units of the Washington DC Veterans Affairs Medical Center voluntarily participated in the initiative between April 2009 and April 2010. Nursing providers were defined as registered nurses (RNs), licensed practicing nurses (LPNs), and clinical nurse assistants (CNAs). Clinical nurse leaders were invited to participate as project champions, and the educational sessions were built into existing educational time. Nurse feedback was continuously elicited, evaluated, and incorporated into the educational sessions. The use of the cumulative unidentified feedback from the nurses and results from anonymous surveys was approved by our Institutional Review Board and the Research and Development Committee. 

### 2.1. Interdisciplinary Team Led Focus Groups and Tag Development

The interdisciplinary QI team consisted of clinical nurse leader project champions, QI educator, and physician champions. At the onset of the project, the QI team organized and led several focus-groups with small groups of nursing providers of approximately 10–12 participants. The focus group format consisted of open-ended questions to explore the role of nurses in catheter care. We engaged stakeholders in eliciting ideas for educational sessions and obtained invaluable perspectives that led to the design of a feasible, easily used catheter label.

**Figure 1:**
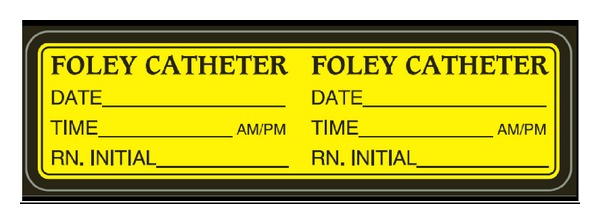
The indwelling urinary catheter (IUC) label design was adapted from intravenous tubing labels, familiar to our nursing staff (manufactured by United Ad Label, RR Donnelly Inc., St. Charles, IL, USA). The IUC tag (3 × 0.9 inches) is water resistant, self-adhesive, and brightly colored. The tags are wrapped around the tubing above the catheter drainage bag.

The IUC label (Figure 1) design was adapted from existing intravenous tubing labels, familiar to our nursing staff (manufactured by United Ad Label, RR Donnelly Inc., St. Charles, IL,USA). The nursing staff was responsible for the tag placement, wrapping each self-adhesive label around the tubing above the catheter drainage bag. Labels were water resistant and recorded date and time of IUC insertion. The fluorescent yellow tags were designed to be clearly visible standing at the patient's bedside. Label placement instructions were posted, and packets of labels were made accessible to nursing staff in medication-treatment rooms and mobile medicine carts. 

### 2.2. Educational Curriculum

We developed a hospitalwide educational curriculum led by the QI team and nursing champions. Five thirty-minute educational sessions were cycled over three daily nursing shifts on our two medical floors, to maximize participation. With the support of nursing leadership, the sessions were built into existing nursing educational time. Participation in the case discussions, interactive segments, and surveys were voluntary for the staff. 

The curriculum introduced the QI process and reinforced use of IUC labels (Table 1). The educational curriculum consisted of five sessions: an introduction to the project goals and IUC tags (Session I), nursing role in CAUTI prevention (Session II), case studies (Session III), hands-on microbiology (Session IV), and an educational review session (Session V). There was a focus on the care of IUCs in the context of a nurse scope of practice and the consequences of CAUTIs. We referenced evidence-based guidelines to develop our didactic presentations; we used case-based scenarios to facilitate discussion and conducted hands-on culturing of the environment. To elicit feedback, time for open discussion was built into the end of each session. The final session featured an educational board game reviewing strategies to reduce CAUTIs through catheter-care management and hand-hygiene guidelines. 

### 2.3. Eliciting Feedback and Monitoring Efforts

Participants anonymously completed paper-based survey after each of the educational sessions. Staff suggestions were directly incorporated into subsequent sessions. The surveys elicited feedback and provided real-time insight on the learning objectives and content using a 3-point Likert-type scale (*Strongly Agree, Moderately Agree,and Disagree*). The queries asked nurses to rate the projects workplace importance, its impact on improving patient care, and opportunities for collaboration between disciplines. Individually unidentifiable responses gauged interest in the QI process, tested provider knowledge, and assessed the performance IUC identification over time. At the end of each block of educational sessions, nurses were asked to self-report their tagging behavior. The proportion of nurses reporting tagging after the introduction of the IUC tags was compared to the last block of educational sessions utilizing two-tailed Chi-square test and accepting a *P* value of <0.05 (SPSS v11, Chicago, IL). 

The QI team reinforced tagging between educational sessions with routine rounds of the medical floors. Promotional pens highlighting initiative goals were distributed that read “Make your work count,” “Tag a tube” and “Let's work together to reduce CAUTIs.” Recognition of individual catheter-tagging efforts was published in monthly nursing newsletters, and results of the initiative were widely promoted at hospital-wide quality and educational fairs. 

## 3. Results and Discussion 

Thirty educational sessions were conducted over a 1-year period. Nursing providers completed 152 surveys; 76.3% were from RNs including LPNs and 31.6% from CNAs. At baseline, a minority of participants reported prior experience in QI: only 36.2% (42/116) of RNs (including RNs and LPNs) and 30.5% (11/36) of CNAs. 

At the end of each block of educational sessions, survey responses were collated. Among those who responded, 98.4% (121/123) felt increased QI awareness over the course of the educational sessions. In addition, 98.6% (142/144) felt the project facilitated teamwork, and 99.3% (151/152) believed that the project was important to their work. Respondents consistently reported that they felt the project was important to patient care. 

**Figure 2:**
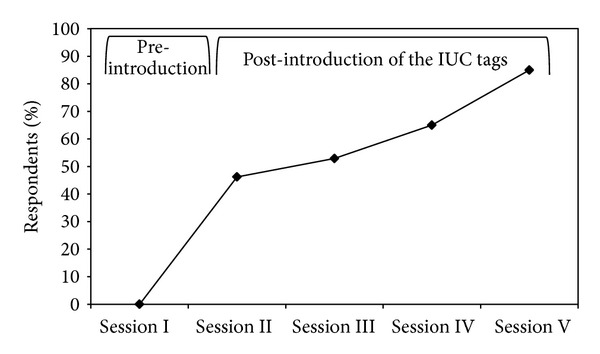
The nursing self-reported catheter tagging increased after the introduction of the IUC tags, rising steadily after each block of educational sessions (Sessions II–V). Once tags were introduced, there was a significant increase in proportion of nurses reporting use of IUC tags at the end of the cycle of educational blocks (from 46.2% to 84.6%, *P* = 0.001).

The nurses self-reported catheter-tagging increase after the introduction of the IUC tags in Session I (Figure 2). In the post-introduction period, we found that there was a significant increase in proportion of nurses reporting use of IUC tags (from 46.2% at the end of Session II to 84.6% at the end of Session V, *P* = 0.001).

In the dissemination of a new program, the innovators and early “adopters” have been implicated in the widespread adaptation of an initiative [11]. From the onset, we targeted nurse providers in the development and implementation of the IUC label consisting of the date of catheter placement. Our carefully designed educational curriculum focused on practical, primarily nurse-controlled risk-reduction interventions. We introduced several novel strategies including practical case-based discussions and fun interactive hands-on culturing of hospital surfaces, including the catheters themselves. The board-game format was popular with staff and easily promoted at hospital-wide events.

## 4. Conclusions

We set out to change behavior through education and renewed CAUTI awareness in the hospital setting. In the process, we collected informal assessments of staff perceptions and self-reported behaviors. Feedback was continuously incorporated, and achievements were recognized. Favorable participant responses and self-reported IUC tagging were early and sustained. 

Changing provider behavior is a challenge across all healthcare settings. We found that the early engagement of nurses and our primary stakeholders, combined with a targeted educational interdisciplinary initiative led to increased awareness of the presence of IUCs, including the concepts of QI. The curriculum resulted in routine adoption of tagging for catheter identification. 

The processes employed in this small scale project can be applied to broader, hospital-wide initiatives and to large-scale initiatives for healthcare interventions. As first line providers with responsibility for the placement and daily maintenance of IUCs, nurses are ideally positioned to implement QI efforts addressing CAUTIs in the hospital setting. 

## Figures and Tables

**Table 1:** Our targeted educational curriculum consisted of five sessions. Each session provided a format of didactic and open discussion components and opportunities for hands-on learning. The participants were inpatient nurse providers, and sessions were led by a QI educator, and nurse project champions.

Session I: introduction	
(i) Review Quality Improvement (QI) principles and practice	
(ii) Review main project goals:	
(a) Increase inpatient provider awareness of catheters	
(b) Increase provider awareness of catheter-associated urinary tract infections (CAUTIs)	
(c) Minimize contamination during clinical sample collection	
(iii) Introduce Indwelling Urinary Catheter (IUC) Tags	
(iv) *Open discussion*: nurses role in addressing CAUTIs	

Session II: nursing role in CAUTI prevention	
(i) Review of the pathophysiology and clinical impact of CAUTIs	
(ii) Review of existing hospital-based Infection Control guidelines	
(iii) Review appropriate techniques for collection of urine culture	
(iv) *Open discussion*: Risk-reduction strategies in catheter care management	

Session III: case-study presentation	
(i) Highlight the clinical impact of CAUTIs, including detailed review of the following:	
(a) Medical indication for catheterization	
(b) Correlation of prolonged catheterization and CAUTI development	
(c) Importance of sample collection in context of culture interpretation	
(ii) *Open discussion:* role of catheter-care in improving health outcomes	

Session IV: hands-on microbiology	
(i) Review basics of sterile technique with emphasis on hand hygiene	
(ii) Introduce microbiologic culture in context of urine sampling	
(iii) Review pathogens commonly associated with CAUTIs	
(iv) Swab hands and hospital surfaces onto 5% sheep blood agar	
(v) *Open discussion*: minimize contamination during clinical sample collection	

Session V: educational review	
(i) Active participation in board-game to review cumulative knowledge of Sessions I–IV	
(ii) *Open discussion*: nurses role in implementing QI efforts addressing CAUTIs	
